# Cerebellar mutism syndrome of non-tumour surgical aetiology—a case report and literature review

**DOI:** 10.1007/s00381-023-05947-8

**Published:** 2023-05-04

**Authors:** Aske Foldbjerg Laustsen, Malene Landbo Børresen, John Hauerberg, Marianne Juhler

**Affiliations:** 1 Department of Neurosurgery, Copenhagen University Hospital Rigshospitalet, Blegdamsvej 9, 2100 Copenhagen, Denmark; 2 Department of Paediatrics and Adolescent Medicine, Copenhagen University Hospital, Rigshospitalet, Blegdamsvej 9, 2100 Copenhagen, Denmark

**Keywords:** Cerebellar mutism syndrome, Posterior fossa syndrome, Arteriovenous malformation, Cerebellar haemorrhage, Postoperative vasospasms

## Abstract

Cerebellar mutism syndrome (CMS) is a well-known complication of posterior fossa (PF) tumour surgery. CMS has previously been reported in cases of non-tumour surgical aetiology in a limited number of publications. We report a case of a 10-year-old girl who suffered a cerebellar haemorrhage and subsequent CMS following surgical treatment of a ruptured arteriovenous malformation (AVM) in the cerebellar vermis. The AVM was removed acutely through a transvermian access, and hydrocephalus was treated with temporary external drainage. In the postoperative period, she suffered diffuse vasospasms of the anterior cerebral circulation and had a permanent shunt placed for hydrocephalus. Her mutism resolved after 45 days but severe ataxia persisted. To our knowledge, this is the first reported case of CMS related to a vermian haemorrhagic stroke with postoperative diffuse vasospasms. Based on this case, we present a literature review on CMS of non-tumour surgical origin in children.

## Introduction

CMS is a well-described complication of PF tumour surgery in children affecting up to 30% of cases. It typically arises within 1–10 days postoperatively [1] and consists of (1) transient mutism or speech reduced to a few words elicited only by vigorous stimulation, (2) emotional lability, (3) ataxia and (4) hypotonia [2]. Preoperative risk factors include tumour size, midline location, brain stem invasion and histopathology of medulloblastoma or atypical teratoid/rhabdoid tumour [1, 3].

Splitting the vermis (transvermian approach) has been suggested as a surgical risk factor of CMS, resulting in recommendations such as vermis-sparring surgical techniques (including the telovelar approach) to reduce the risk. Conflicting results have been published on this matter [4, 5].

It is broadly acknowledged that the dentate-thalamo-cortical pathway (DTCp) plays a central role in the development of CMS explained by proximal damage (either direct surgical trauma or subsequent postoperative oedema) to the dentate nucleus (DN) or superior cerebellar peduncle (SCP) leading to a disruption of the pathway resulting in cerebello-cerebral diaschisis and localized supratentorial hypoperfusion [6].

Traditionally, CMS has been related to PF tumour surgery; however, non-tumour surgical cases have been described including PF stroke, traumatic brain injury, cerebellar infection and inflammation as well as metabolic diseases [7–46]. A vascular aetiology like vasospasms underlying CMS has previously been hypothesized but not substantiated [47].

The aim of this study is to present a case of CMS following a haemorrhagic stroke from an AVM in the vermis in a paediatric patient. As the literature on CMS related to non-neoplastic pathologies is scarce, we additionally conducted a comprehensive literature review of paediatric cases of CMS of non-tumour surgical origin.

## Case report

**Fig. 1:**
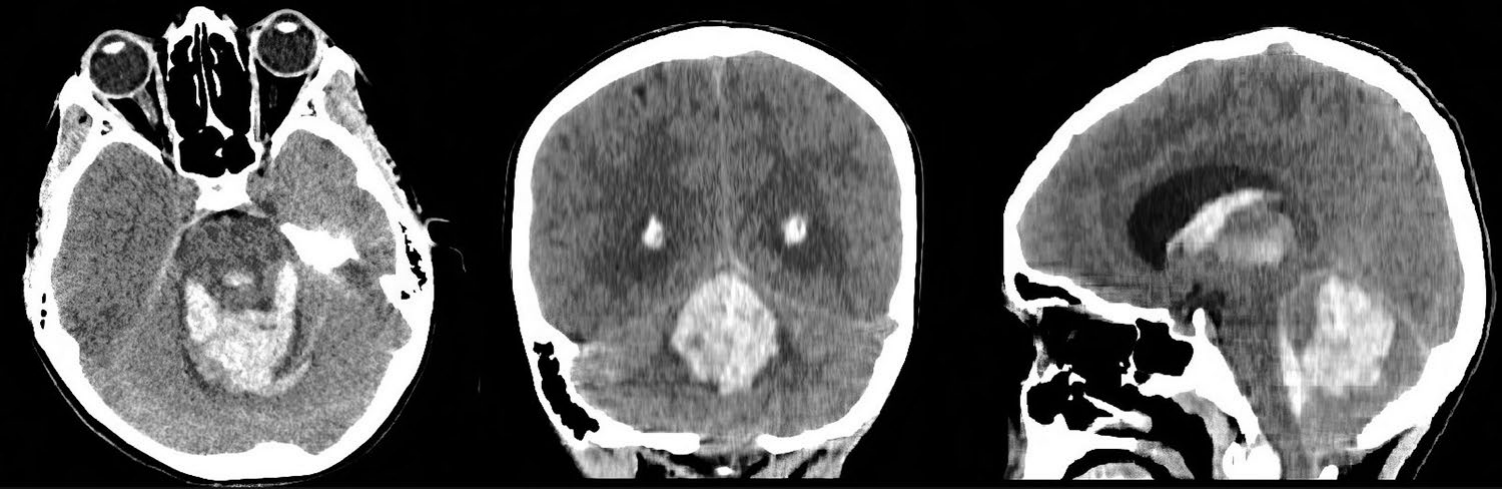
Primary CT scan

We present a 10-year-old girl, right-handed with unremarkable health and developmental history, who while watching TV suffered a sudden onset of frontal headache, quadriparesis and explosive vomiting followed by seizuring. She lost consciousness, was intubated and rushed to the hospital. On admission, she had isochoric pupils with normal light reflex. A computed tomography (CT) scan was performed showing a haemorrhagic lesion of the PF involving vermis with perforation to the ventricular system and hydrocephalus (Fig. 1).

**Fig. 2:**
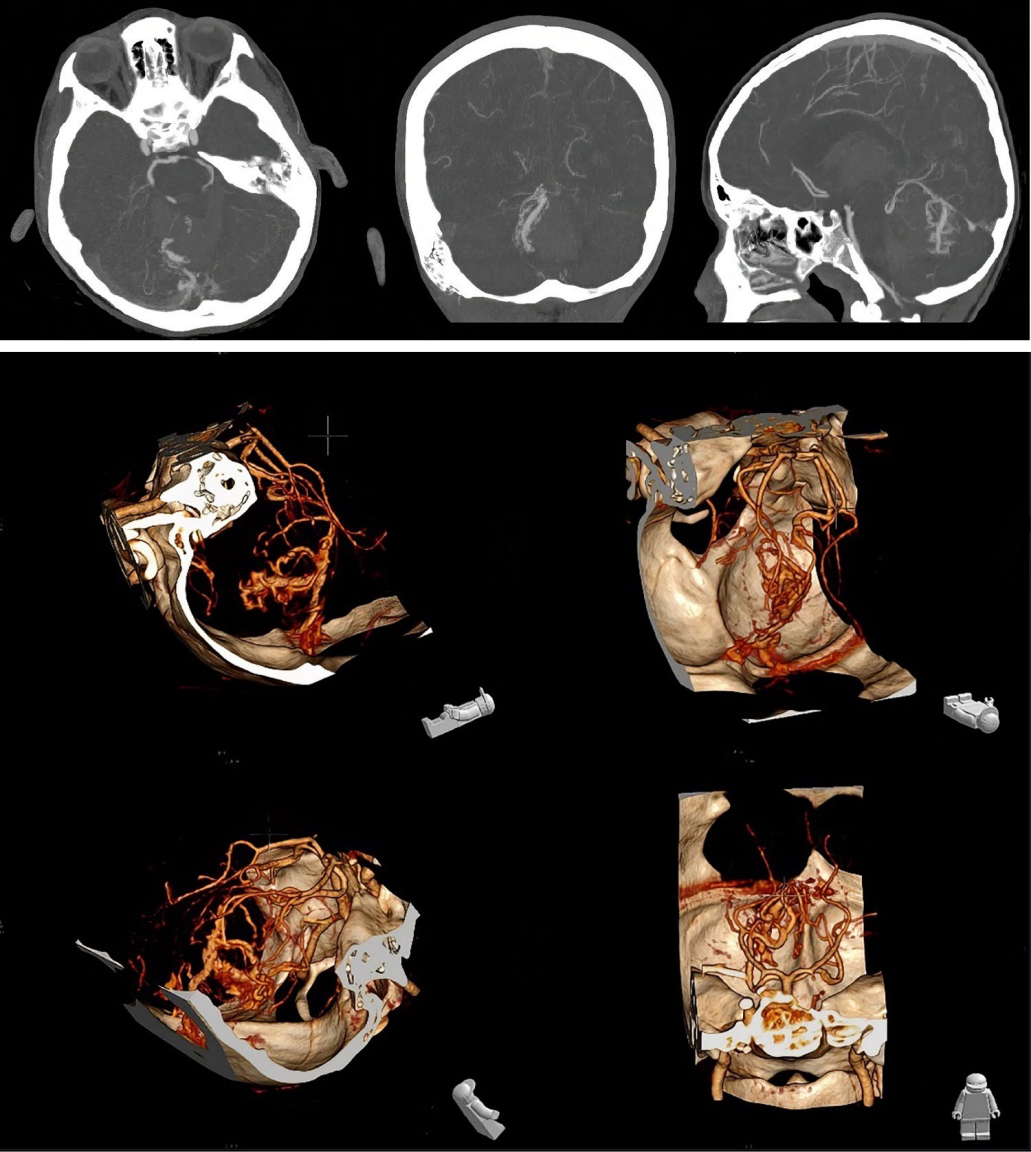
CT angiography with 3D reconstruction of the AVM

The CT angiogram revealed an AVM in the vermis. The AVM was supplied by branches from both superior cerebellar arteries and classified as Spetzler-Martin grades 2–3 (Fig. 2).

Surgery was performed immediately after admission with the removal of the haematoma and AVM through a transvermian access. Hydrocephalus was initially treated with external ventricular drainage (EVD).

The girl was extubated on the first postoperative day uttering single words or short sentences. She had ataxia of the upper extremities and ocular motor control difficulties. She spoke few words on the 3rd postoperative day and underwent a postoperative digital subtraction angiography (DSA), which showed no sign of residual AV shunting.

On day 4, she had full onset mutism accompanied by agitation and emotional irritability. Her auditory and verbal comprehension was deemed intact as she was able to respond adequately to questions by squeezing either her right or left hand.

**Fig. 3:**
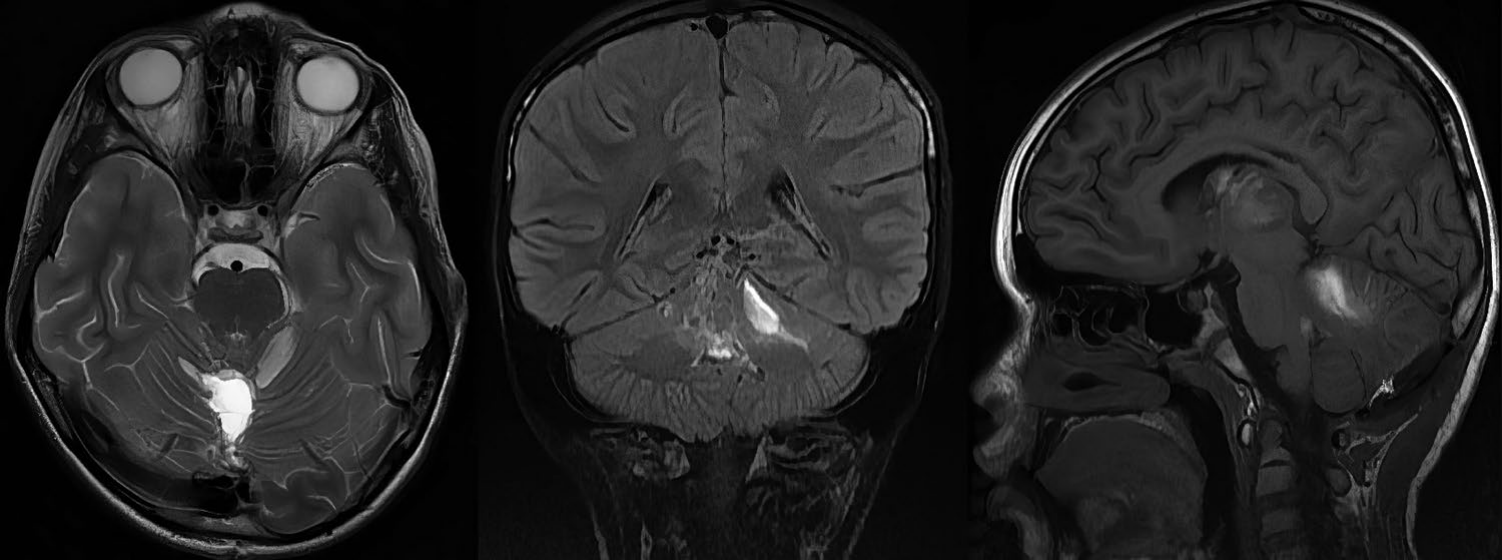
MRI findings from day 11

A follow-up magnetic resonance imaging (MRI) of the brain on the 11th postoperative day showed oedema and haematoma involving both superior cerebellar peduncles (SCP) and vermis, a small haematoma remnant in the surgical cavity and perifocal oedema surrounding the cavity (Fig. 3).

Her EVD was removed on day 17. Increased agitation was observed in the following days. On day 19, a CT cerebral angiography was performed due to sudden onset of right upper extremity paralysis showing narrowing of the anterior cerebral artery (ACA) and median cerebral artery (MCA) bilaterally as well as hydrocephalus. A diagnosis of vasospasms was suggested but not confirmed on a DSA. Furthermore, there was doubt whether the possible vasospasms were in fact the cause of her localized paresis. There was no angiographic evidence of AVM remnants. Hydrocephalus was treated with reimplantation of an EVD and replaced by a ventriculo-peritoneal (VP) shunt 3 days later. She was transferred to the neurointensive care unit for conservative treatment of vasospasms with calcium antagonists (nimodipine 1 g/kg × 6) for 7 days. A follow-up CT cerebral angiography on day 20 showed no signs of vasospasms. Due to dysphagia, she had a gastrostomy tube inserted on day 26. On day 33, she was transferred to a specialized neurorehabilitation unit. Her right upper extremity paresis had resolved but she suffered from global hypotonia.

Her mutism lasted 45 days and on day 49 she started producing single words. On day 77 she was discharged to outpatient rehabilitation, with ongoing symptoms of ataxic dysarthria and dysprosody. Prior to discharge, her neuropsychological evaluation with WISC-V (Wechsler Intelligence Scale for Children, 5th edition) and TOMAL-2 (Test of Memory and Learning, 2nd edition) revealed reduced verbal working memory and learning ability.

At 4-month follow-up, she was able to speak in full sentences, however still dysarthric. Furthermore, she suffered from severe upper limb ataxia and was dependent on a walker. The last follow-up was 12 months post ictus where cognition and language were re-established; however, she suffered from marginally slowed speech and slight dysarthria. She had ataxia and impaired balance primarily on the right side. She was able to write with her right hand, although compromised, and ate without dysphagia.

## Literature review

### Methods

A PubMed literature search until November 31, 2022, was conducted. The search generated 347 articles. The following MeSH search term was used: (((“Mutism”[Mesh] OR Mutism OR mute)) AND (“Adolescent”[Mesh] OR “Child”[Mesh] OR “Infant”[Mesh] OR Adolescent OR Child OR Infant OR Paediatric OR Pediatric)) AND ((“Cerebellar Diseases”[Mesh] OR Cerebellar OR Posterior Fossa)))).

**Fig. 4:**
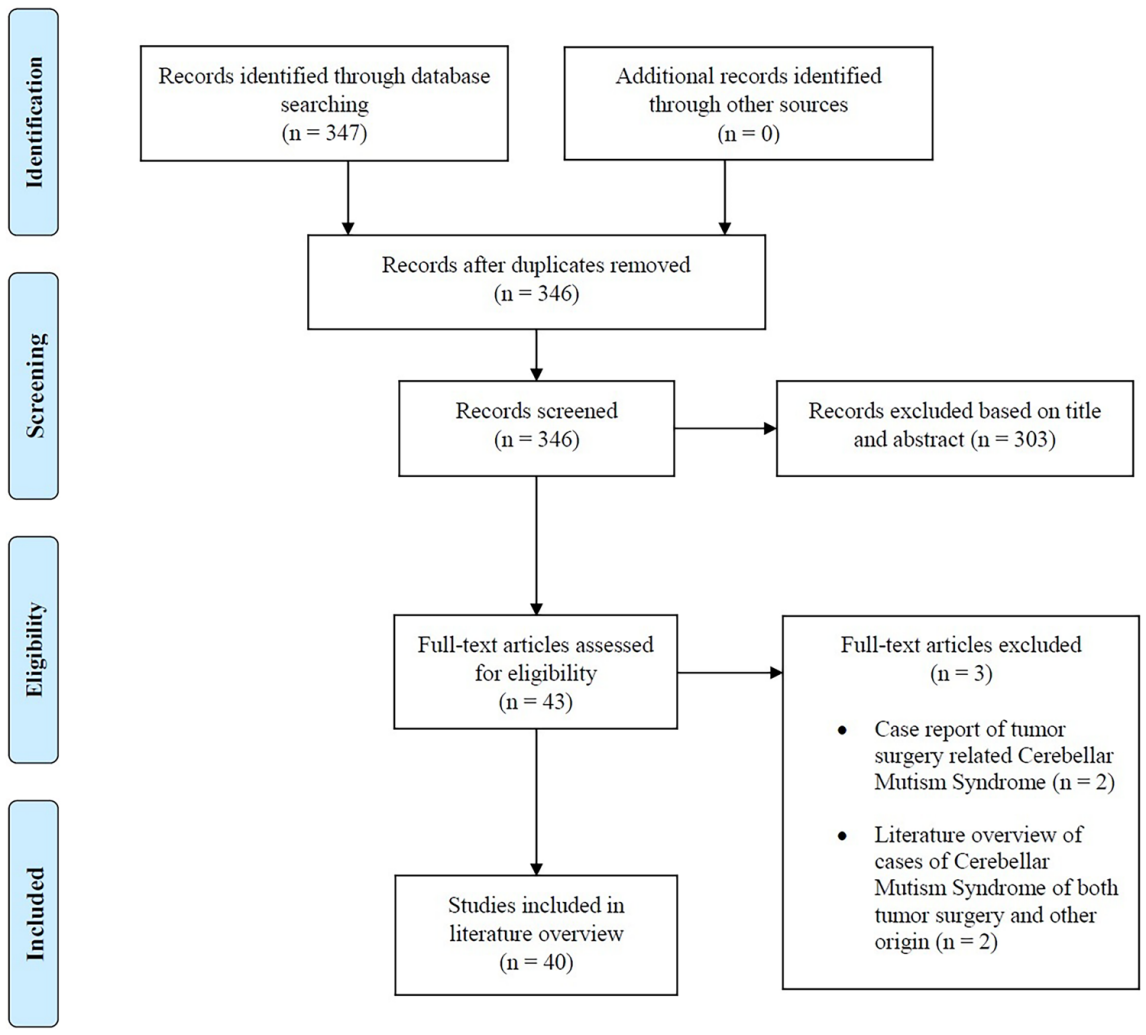
Prisma diagram over literature search

Inclusion required the following: (1) mutism of cerebellar origin, (2) no cerebellar tumour surgery, (3) paediatric patients defined as < 18 years at diagnosis. All titles and, when relevant, abstracts and full articles were reviewed by the first author. Forty case reports were included in the literature review and previous relevant focused literature reviews were included as references in the discussion. Cross-checking references from included case reports yielded no additional relevant articles. Figure 4 shows a prisma diagram over the literature search and inclusion.

## Results

Table 1 summarizes the clinical and paraclinical relevant findings of the case reports. Table 2 gives an overview of the demographics, predominant clinical and neuroradiographic features of the 3 largest case groups.

**Table 1:** Summary of objectives and findings in included case reports

Study (year, language if not English)		Age (years)/sex (F/M)	Aetiology		Mutism duration (days)	Other relevant symptoms (as described in the case report)		Relevant radiological findings (MRI unless stated otherwise)	Last follow-up (months)	Persisting neurologic deficits		Surgery
Shiihara et al. (2007)		2.6/F	Cerebellitis from rotavirus		< 148	Wide-based gait, hypotonia		Initially non, at final follow-up cerebellar atrophy and enlarged 4th ventricle	5.3	Slow speech, dysarthria, right-hand tremor		None
Takanashi et al. (2010)		2/M	Cerebellitis from (assumed) rotavirus		19	Ataxia, dysarthria*		Lesion in vermis, atrophy	1	Ataxia and dysarthria		None
		2/F	Cerebellitis from rotavirus		4	Ataxia, dysmetria, dysarthria*		Lesion in vermis, atrophy	1	Dysarthria		None
		2/F	Do		10	Ataxia, dysarthria*		Lesion in vermis, atrophy	1.4	Ataxia, mental retardation		None
		3/F	Do		9	Ataxia, dysarthria*		Lesion in vermis, atrophy	2	Ataxia, dysarthria		None
		3/F	Do		11	Hypotonia, dysmetria, dysarthria*		Lesion in vermis, atrophy	1	Dysarthria, tremor		None
		3/F	Do		11	Ataxia, tremor, dysarthria*, seizures		Lesion in cerebellar nuclei, atrophy	2	Dysarthria		None
		3/M	Do		3	Dysarthria*, ataxia		No relevant lesions	1	None		None
		3/M	Do		15	Ataxia, dysarthria*		Lesion in vermis, atrophy	1.5	Dysarthria		None
		4/F	Do		12	Seizures, hypotonia, ataxia, dysarthria*		Lesion in vermis, atrophy	1	Dysarthria		None
		4/F	Do		17	Seizures, hypotonia, ataxia, dysarthria*		Atrophy, no lesions	12	Ataxia		None
Kubota et al. (2011)		3.2/M	Cerebellitis from rotavirus		34	Generalized ataxia and intentional tremors		Initially hyperintensity changes in bilat. DN, follow-up MRI with diffuse cerebellar atrophy	5	Dysmetria, slurred/explosive speech		None
		2.3/F	Do		17	Dysarthria*, wide-based gait, truncal ataxia, intentional tremor		Initially hyperintensity changes in bilat. DN, vermis and CH. Follow-up MRI with slight atrophy of cerebellum	6	Slurred/explosive speech		None
Engan et al. (2016, Norwegian)		4/F	Cerebellitis from rotavirus		14	Global hypotonia and dysmetria		Oedema and reduced diffusion in DN bilat	6	Slow, monotonous speech, fine motor skill problems		None
Paketci et al. (2020)		4/F	Cerebellitis from rotavirus		< 112	Truncal instability, dysarthria*, hypotonia		Reduced diffusion in DN bilat	4	Slow and dysarthric speech, poor fine motor skills		None
Fluss et al. (2010)		2.5/F	Cerebellitis from influenza A virus		5	Ataxia, dysarthria*, word-finding difficulties		Restricted perfusion of right DN	1	None		None
Sanders et al. (2020)		10/M	Acute necrotizing encephalopathy from influenza A virus		< 180 (diminished speech)	Emotional instability, behavioural problems		Hypodensities in thalamus, diffuse oedema and haemorrhagic lesions of cerebellum, brain stem compression	6	None		EVD^**^
Thabet et al. (2013)		3/M	Encephalopathy from influenza B virus		< 42	Seizures, global hypotonia		Oedema of middle cerebral peduncles and bilat. DN	1.5	Dysarthria, flat-fooded gait, phonological impairment		None
Erol et al. (2013)		7/F	Cerebellitis from varicella-zoster virus		28 (anarthria)	Unsteady gait, global hypotonia, nystagmus, truncal ataxia, tremor		MRI unremarkable	2	None		None
Brito et al. (2019)		6/F	Cerebellitis from varicella-zoster Virus		7	Unsteady gait, ataxia, inappropriate laughter		None	1	Dysarthria		None
Drost et al. (2000)		4/F	Pneumococcal meningitis		3	Dysarthria*		Cerebellar swelling with surface lesions, subdural hygroma over left hemisphere	2	None		None
Riva et al. (1998)		4.2/F	Cerebellitis of unknown aetiology		28	Ocular dysmetria, ataxia, reduced consciousness		Cerebellar oedema	9	Cognitive deficit, aphonic/aprosodic speech		VP^***^ shunt
Mewasingh et al. (2003)		5/F	Cerebellitis following colitis		10–14	Dysarthria*, ataxic gait, hypotonia, dysmetria		Cerebellar hyperintensity changes	0.9 (mortem)	-		None
		4/F	Do		21	Dysarthria*, upper limb dysmetria, ataxic gait		Cerebellar atrophy	12	Visuospatial difficulties		None
Papavasiliou et al. (2004)		3/F	Cerebellitis of unknown aetiology		14	Dysmetria, tremor, truncal ataxia, dysarthria*		Atrophy of vermis	18	Ataxic gait, dysarthria, dysmetria		None
Dimova et al. (2009)	7.5/F		Cerebellitis of unknown aetiology		< 180	Truncal instability, hypotonia, dysmetria, pathologic laughter, ataxia		Cerebellar hyperintensity changes followed by cerebellar atrophy	6	Dysmetria, speech impairment		None
Parrish et al. (2009)	2.2/M		Acute disseminated encephalomyelitis of unknown aetiology		< 3	General weakness, inability to walk		Hyperintensity signal changes of DN and SCP bilat	6		Impaired speech	None
	2.5/M		Do		2	Gait impairment, ataxia, altered mental status, aphasia		Oedema of CH, hyperintensity signal changes in deep cerebellar nuclei	36		Ataxia, cognitive delay, limited speech, behavioural problems	None
	2.6/M		Do		28 (limited speech)	Unsteady gait, irritability		Hyperintensity signal changes in brachium pontis surrounding DN bilat	30		Dysmetria, speech delay, dysarthria	None
McAndrew et al. (2014)	7/M		Acute disseminated encephalomyelitis of unknown aetiology		6	General weakness, dysmetria		Sinus vein thrombosis in superior sagittal sinus and right sigmoid confluence, diffusion restriction in bilat. DN and right CH	2		Cognitive impairment, dysprosody, word-finding difficulties	None
Makarenko et al. (2018)	9/F		Cerebellitis of unknown aetiology		N/A	Unconsciousness from hydrocephalus		Diffuse bilat. Cerebellar oedema, compression of 4th ventricle, tonsillar crowding of foramen magnum	N/A		Speech impairment	EVD^**^
Barragan-Martinez et al. (2017, Spanish – abstract only)	2 or 4 (sex N/A)		Parainfectious acute cerebellitis (aetiology N/A)		N/A	N/A		N/A	N/A		Mild speech alteration	N/A
Niedermeijer et al. (2015)	13/F		Late-onset OTC^****^-deficiency		4	Dysarthria*, bilat. limb ataxia, axial ataxia, behavioural changes		None	1.5		Mild slow speech disturbance	None
Frassanito et al. (2009)	7/F		Spontaneous intratumoural haemorrhage		10	Gait instability, severe dysarthria, tremors of extremities and head		Tumour arising from quadrigeminal plate, peritumoural bleed in the upper vermis	None		Mild dysarthria	Tumour resection after 8 weeks
Sinha et al. (1998)	8/M		Spontaneous haemorrhagic stroke in vermis		42	Urinary retention, inability to eat or swallow (normal gag reflex)		Vermian haematoma without vascular abnormality	9		Dysarthria	Immediate surgical evacuation
Frim et al. (1995)	8/F		Cavernous malformation in right pons and SCP		12	Left-sided hemiparesis, dysarthria*		Cavernous malformation in right pons at level of MCP with haemorrhagic component	4		Minimal hemiparesis	Immediate surgical evacuation
Wang et al. (2002)	14/M		Cavernous malformation in mesencephalon		12	Dysarthria*, dysmetria, cranial nerve impairment (L-III, R-V, R-VII)		Brainstem cavernous malformation and related haematoma. Postop. with oedema of brainstem	7		None	Excision 7 days post ictus
Miyakita et al. (1999)	3.7/F		Brain stem infarct from traumatic vertebral artery injury		10	Right hemiplegia, upward gaze palsy		Right vertebral artery dissection (angiography). Lesion in tegmentum of lower left midbrain to upper pons and left cerebral peduncle	2		Right-hand weakness	None
Kossorotoff et al. (2010)	3.4/M		Vertebral artery dissection and basilar artery occlusion		N/A	Dysarthria*, emotional lability		Stroke in the left pons	N/A		Hemiparese, writing difficulty	None
	8/M		Left vertebral artery dissection		N/A	Mood instability, outburst of laughter or crying		Stroke in right CH, vermis and bilat. in the brainstem	N/A		Dysmetria, writing difficulty	None
	9.7/M		Basilar artery occlusion		N/A	Agitation, outburst of laughter or crying		Stroke in the right pons	N/A		Writing difficulty	None
Hashem et al. (2021)	2/M		Hyperleukocytosis-induced stroke from T cell acute lymphoblastic leukaemia		35	Altered mental status, hypotonia		Multifocal infarctions in left pons, bilat. CH and tonsillar herniation	6		Significant improvement in neurologic status	None
Dietze et al. (1990)	15/F			Paravermian AVM	84	Diffuse hypotonia, appendicular ataxia, truncal ataxia		PF haematoma extending into both CH	3		Dysarthria, truncal ataxia	Immediate surgical removal
Vandeinse et al. (1996)	11/M			PF AVM	19		Dysarthria*, emotional lability, minimal awareness	N/A	N/A		N/A	Surgical removal, VP*** shunt
Al-Anazi et al. (2001)	14/F			Vermian AVM	35		Ataxic speech	Vermian haemorrhage with oedema	6		None	Embolization 6 weeks post ictus
Turkel et al. (2003)	2.8–15.7 (sex N/A)			PF AVM	N/A		Behavioural symptoms	Posterior fossa AVM with affection of left CH	N/A		N/A. 2 relevant patients described in case series	Surgical removal
Baillieux et al. (2007)	12/F			Vermian AVM	4		Behavioural changes, right-sided ataxia	Haemorrhage in vermis, destruction of DN and involvement of right cerebellar hemisphere (CT)	6		None	Surgical removal 2 weeks post ictus
Demartini et al. (2020)	17/F			Vermian (culmen) AVM	> 180		Right-sided tremor	Haemorrhage in vermis and 4th ventricle. Follow-up MRI with gliosis and encephalomalacia	6		Dysmetria, gait ataxia, tremor, dysdiadochokinesia.	Immediate embolization
Koh et al. (1997)	6–12 (sex N/A)			PF AVM/PF trauma	10–56		N/A	Paramedian AVM in vermis and adjacent CH/contusion in left CH after traffic accident	N/A		N/A. 2 relevant patients described in case review	Surgical removal
Ersahin et al. (1996)	2.5/M			Traumatic paravermian haematoma	56		Ataxia	PF haematoma in the right paravermian region (CT)	14		Dysarthria (until 8 months)	None
Fujisawa et al. (2004)	7/M			Traumatic subdural PF haematoma	14		Ataxia	Acute subdural haematoma of right PF, traumatic subarachnoid haemorrhage in left Sylvian cistern and brain stem distortion (CT). Follow-up MRI with ischaemic lesions in pons and cerebellum	2.6		Mild ataxia	Immediate evacuation of haematoma
Kariyattil et al. (2015)	7/M			Traumatic cerebellar cortical haematoma	14		Right facial nerve palsy, weakness of upper limbs	Comminute fracture of right occiput, cerebellar contusion and 4th intraventricular haemorrhage (CT)	8		None	None
Lahirish et al. (2021)	8/F			Penetrating trauma to PF	6		Right facial nerve palsy, torticollis, horizontal nystagmus, impaired finger-nose and dysdiadochokinesia	Skull base fracture, right cerebellar contusion and oedema (CT)	1		Right facial palsy	None
Chivet et al. (2022)	5.3/F			Isolated traumatic cerebellar injury	12		Dysarthria*, apathy, irritability, hypotonia	Injury in vermis, bilat. CH, 4th ventricle and bilat. DN	11		Concentration difficulties	None
	2.6/F			Do	Few		Dysarthria*, apathy, hypotonia	Injury in unilateral CH, tonsil, 4th ventricle and DN. Right-sided occipital fracture	72		None	None
Yokota et al. (1990, Japanese – abstract only)	6/M			Head trauma to the left temporooccipital region	N/A		N/A	Contusion of vermis and left CH	N/A		N/A	N/A

*DN* dentate nucleus, *SCP* superior cerebellar peduncle, *MCP* middle cerebral peduncle, *CH* cerebellar hemisphere, *Bilat*. bilateral, *N/A* not available, *PF* posterior fossa, *AVM* arteriovenous malformation

^*^Dysarthria subsequent to mutism

^**^External ventricular drain

^***^Ventricular-peritoneal shunt

^****^Ornithine transcarbamylase

**Table 2:** Overview of 3 largest groups of cases of CMS in the literature (all values are estimated from the available information in the included case reports)

Aetiology	Case reports (*N*)	Number of patients (*N*)	Mean age in years (range)	Median duration of mutism in days (range)	Summary of predominant accompanying symptoms	Summary of predominant structural damage (verified by neuroradiography)	Mean follow-up in months (range)
Cerebillitis from infection	13	25	3.9 (2 to 9)	14 (3 to < 180)	Dysarthria, ataxia, hypotonia	Lesion of vermis, lesion of unilat. or bilat. DN, cerebellar atrophy	4.4 (0.9 to 18)
AVM	7	8	13.8 (11 to 17)	29 (4 to > 180)	Ataxia, behavioural changes, dysarthria	Haemorrhage in vermis, haemorrhage in unilat. or bilat. CH	5.3 (3 to 6)
TBI	7	8	5.5 (2.5 to 8)	14 (6 to 56)	Ataxia, dysarthria, hypotonia	Unilat. or bilat. Cerebellar contusions, lesion in vermis, IVH in 4th ventricle	18.1 (1 to 72)

*DN* dentate nucleus, *AVM* arteriovenous malformation, *CH* cerebellar hemisphere, *TBI* traumatic brain injury, *IVH* intraventricular haemorrhage

Included publications contained a total of 58 reported cases divided into groups depending on the probable or known cause of cerebellar mutism.

Mutism ranged from 2 days to > 180 days with a median of 14 days; however, some articles did not report the exact duration of mutism. Predominant accompanying symptoms involved limb or truncal ataxia, behavioural changes and hypotonia, as well as subsequent ataxic dysarthria. Predominant persisting neurological deficits at the final follow-up involved dysarthria, slow speech and gait or limb ataxia with follow-up ranging from 2 weeks to 6 years. One case died after 26 days from the primary incident, 2 articles did not include follow-up in the history and 5 articles did not report follow-up time.

### Case reports of CMS related to infection

The most frequently reported causes were cerebellitis due to rotavirus (*n* = 17 in 5 case reports [16, 27, 34, 39, 41] followed by cerebellitis or encephalopathy from influenza virus A/B (*n* = 3 [19, 38, 42]), cerebellitis from varicella-zoster virus (*n* = 2 [10, 17]) and pneumococcal meningitis (*n* = 1 [15]). In 10 cases (8 case reports [9, 14, 29–31, 35–37]), cerebellitis and acute disseminated encephalomyelitis were described without an identified pathogen.

Duration of mutism lasted from 2 days to < 180 days. Two cases had hydrocephalus treated with EVD and 1 case was treated with a VP shunt. Case information from one article in Spanish was included only from the abstract.

### Case reports of CMS related to haemorrhagic vascular pathology

#### AVM

Eight patients in 7 case reports were identified [7, 8, 12, 13, 25, 43, 44]. In all cases, the AVM was located in the vermis. All cases presented with a haemorrhage from the AVM. In 6 patients the AVM was surgically removed and in 2 patients the AVM was treated with embolization. One patient was treated with a VP shunt. None reported postoperative or posthaemorrhagic vasospasms.

Mutism lasted from 4 days to > 180 days (mute at last follow-up) and 1 article did not account for the duration of mutism.

#### Cavernous malformation

Two reports each with a single case describe CMS related to a cavernous malformation [21, 45], the first case located in the right pons and SCP and the second case located in the mesencephalon. Both cases presented with haemorrhage from the cavernoma. One case had immediate surgical removal of the cavernoma and 1 patient had surgical removal of the cavernoma 7 days post ictus. The duration of mutism was 12 days in both cases.

#### Haemorrhagic stroke

One case report described mutism in a single patient after suffering a spontaneous haemorrhagic stroke in the vermis [40] without apparent vascular abnormality. The haematoma was immediately evacuated. Mutism lasted 42 days.

#### Spontaneous intratumoural bleed

A single case reported mutism from a spontaneous intratumoural haemorrhage lasting a total of 10 days [20]. The tumour was surgically removed 8 weeks after the haemorrhage.

### Case reports of CMS related to ischaemic vascular pathology

Five patients suffering mutism following a vascular ischaemic stroke were described in 3 case reports [23, 26, 32]. Three cases suffered ischaemic stroke subsequent to vertebral artery dissection, of which 1 was caused by trauma. One case suffered isolated spontaneous basilar artery occlusion and finally 1 patient suffered hyperleukocytosis-induced stroke during the onset of ALL. All were treated conservatively. The duration of mutism lasted from 10 to 42 days, with 1 article not accounting for the duration of mutism.

### Case reports of CMS related to TBI

Eight patients in 7 case reports were identified [11, 18, 22, 24, 25, 28, 46]. One patient had immediate evacuation of an acute subdural haematoma in the PF; the remaining cases were either treated conservatively (5/8) or treatment was unaccounted for (2/8). Mutism lasted from a few days (without further specification) to 56 days. One included case was reported in Japanese with an English abstract with no documentation of mutism duration.

### Other pathologies

We found one case of reported mutism related to a metabolic disorder (ornithine transcarbamylase deficiency). This X-linked urea cycle disorder results in neurotoxic hyperammonaemia with neurologic symptoms such as seizures, decreased consciousness and abnormal motor function [33]. In this reported case mutism lasted 4 days.

## Discussion

This case of CMS in a 10-year-old girl following emergency surgery for a ruptured vermian AVM and the additional literature review support alternate aetiologies other than cerebellar tumour surgery to potentially result in CMS.

In our literature search, we encountered a few reviews on subgroups, but no previous comprehensive literature review of paediatric cases of CMS of non-tumour surgical origin. Baillieux et al. [8] reviewed the literature on CMS of *vascular origin* in 2007 and found 10 published cases. Makarenko et al. [29] reviewed literature on CMS *of non-surgical origin* in 2017 finding 20 patients. Lahirish et al. [28] reviewed the literature on CMS in paediatric *head trauma* in 2021 and included 6 patients. Thus, the current literature review contributes with an updated, comprehensive and collected overview for future reference. An important note is that the majority of case reports (*n* = 30/40) included in this literature overview were published prior to the Delphi Consensus definition of CMS [2] potentially leading to case heterogeneity as the definition of CMS was not consistent.

The anatomical substrate of CMS is broadly acknowledged to be a cerebello-cerebral efferent pathway, namely the DTCp, where proximal damage to the tract results in diminished cerebellar input to the cerebral cortex. Additionally, recent research has suggested that the fastigial nucleus and the periaqueductal grey matter may play an underappreciated role in this syndrome [48]. Numerous theories of the pathophysiology of DTCp disruption have been suggested. The main theory is that the surgical approach may cause direct injury to the DN or SCP. Other theories involve postoperative oedema affecting regions of interest, axonal injury and degeneration due to a cytotoxic environment, thermal injury related to the use of an ultrasonic surgical aspirator and cerebellar perfusion deficits due to vasospasms [47]. Potentially, tissue damage involving the same regions due to either primary cerebellar insults or cerebellar surgery for other pathologies than neoplasia could also trigger pathophysiologcal mechanisms hypothesized for CMS. The potential role of hydrocephalus in CMS risk and severity has yet to be substantially elucidated.

The reason for CMS in our case is likely to be multifactorial supporting the theory of CMS originating from various pathophysiological mechanisms. Our patient suffered tissue trauma—both from the stroke and surgery—to presumed critical structures of the syndrome; her imaging showed blood and oedema involving SCP and the deep cerebellar structures bilaterally. In addition, she had possible diffuse vasospasms of the anterior cerebral circulation and suffered from hydrocephalus, treated initially with an EVD and then with a permanent shunt after a failed attempt to relieve her from the EVD.

### Postsurgical CMS

Our comprehensive literature review supports the theory of CMS being due to damage of anatomical substrates, regardless of aetiology, rather than being a direct consequence of PF tumour surgery. Concomitantly, several cases reported CMS after non-tumoural PF surgery, both endovascular [7, 12] and open resection [13, 21, 22, 40, 45]. It has previously been suggested that a vermis-sparing, telovelar approach to the 4th ventricle reduced the risk of CMS. However, a prospective study on 500 children with PF tumours found no difference in the risk of postoperative speech impairment between the telovelar and transvermian approach [1]. CMS in the presented case occurred after applying a transvermian access for evacuating the haematoma and excising the AVM, with a delayed onset similar in time to CMS following PF tumour surgery. In our case, the postoperative MRI on day 11th revealed oedema and blood in the bilateral SCP. Interestingly, studies report conflicting results on lateralized damage to the SCP and risk of CMS; however, bilateral damage to the SCP as in our case seem to increase the risk [49]. As in previously published cases, it is difficult to determine whether the injury from the initial haemorrhage, the subsequent surgical evacuation or a combination of the two caused CMS in our case.

During the admission, our case experienced a sudden onset of right upper extremity paralysis with a CT cerebral angiography suggestive of vasospasms, however not confirmed by DSA. A CT cerebral angiography the following day showed normalization of the artery caliber. Diffuse vasospasms in PF tumour surgery have previously been reported in cases [47] but to our knowledge not in context with paediatric PF AVM resection. A recent case from Deghedy et al. showed basilar artery spasm and subsequent mutism in a patient who underwent PF tumour resection 3 days prior to the insult. Thus, CMS due to vasospasms of the microcirculation seems plausible due to the syndrome’s delayed onset from surgery, yet no substantial study supporting this hypothesis has been published. Treatment with a calcium antagonist was applied to alleviate the diffuse vasospasms in our case, although mutism persisted long after the therapy was stopped. In our opinion, it is speculative yet interesting whether calcium antagonists could alleviate symptoms of CMS if vasospasms prove to play a central part in the aetiology of CMS.

No substantial pharmacological treatment of CMS has been published. Fluoxetine, zolpidem, bromocriptine and donepezil have been suggested but only reported in a small number of cases [50]. Although, a recent study using metformin to promote neurogenesis showed promising results on neurocognitive outcome [51], current  suggested treatment is limited to rehabilitation of speech, neurocognition and motor skills.

### CMS following non-surgical cerebellar insults

Our review reveals that several cases of CMS of non-surgical aetiology have been published, ranging from infection and subsequent cerebellitis over spontaneous intratumoural bleeding and stroke-induced to direct cerebellar trauma. Predominant radiological findings included lesions in the vermis and diffusion restriction in DN. A study by Di Rocco et al. [52] on 34 children with PF tumours found preoperative language impairment in 11 patients, suggestive of a subclinical state of CMS even before surgical intervention. Furthermore, the subgroup with preoperative language impairment had a higher incidence of tumour invasion of the DN. The involvement of the DN is in accordance with the current understanding of the anatomical substrates of CMS. It seems conspicuous that multiple incidents can cause CMS, surgical trauma being just one of many. In our opinion, it is crucial to understand the phenomenon that we move beyond defining CMS as a postoperative complication to PF tumour surgery.

### CMS severity related to AVM

Even though no analytical statistics were applied in this article, there may be a tendency toward mutism duration lasting longer in AVM cases. The median duration of mutism was 29 days in the AVM group, whereas the median duration of mutism in the TBI and cerebellitis group was 14 days corresponding well with the median duration of 16 days found in the study of PF tumours by Grønbæk et al. [1]. Perhaps this difference could be explained by both the spontaneous bleeding and the surgical trauma causing additive injury to the cerebellum and related deep structures, thus amplifying the impact. Furthermore, it is likely that only the most severe cases generate published case reports.

### Neuropsychological consequences of CMS

Mutism is the most apparent syndrome of CMS, but long-term neuropsychological consequences in children suffering from PF tumours with subsequent CMS have gained increased focus in the recent years. In our case, the patient had a remarkable performance in school prior to the ictus according to her parents. During her in-hospital rehabilitation around day 75 postoperatively, TOMAL-2 and WISC-V showed reduced verbal working memory and reduced learning ability suggesting long-term neuropsychological problems as consequences of her disease. Children with CMS seem to experience a significant decline in their intellect [53], although long-term neuropsychological deficits of PF surgery and CMS are currently scarcely elucidated and possibly underappreciated. A recent study on the rehabilitation of children treated for PF tumours [54] concluded that advancements in computer technology and digital tools have led to targeted rehabilitation of neurocognitive deficits. However, future studies evaluating treatment and rehabilitation protocol efficacy are needed.

## Conclusion

This case presentation along with the accompanying literature review provides evidence that CMS can be of non-tumour surgical origin, symptoms being similar to CMS related to PF tumour surgery. Furthermore, our presentation suggests that a broader range of pathologic factors than previously acknowledged may have the potential to cause CMS including a potential role of vasospasms. Clarifying such factors could point at treatment options to alleviate symptoms. In this way, PF pathology of non-tumoural aetiology may be helpful in explaining this severe and devastating syndrome.

## Data Availability

Not applicable.
